# The statistical evidence missing from the Swedish decision-making of COVID-19 strategy during the early period: A longitudinal observational analysis

**DOI:** 10.1016/j.ssmph.2022.101083

**Published:** 2022-03-31

**Authors:** Xiaoqin Wang, Fan Yang Wallentin, Li Yin

**Affiliations:** a Department of Electrical Engineering, Mathematics, and Science, University of Gävle, Sweden; b Department of Statistics, Uppsala University, Sweden; c Department of Medical Epidemiology and Biostatistics, Karolinska Institutet, Sweden

**Keywords:** COVID-19, Decision-making, Longitudinal analysis, Statistical evidence, Swedish strategy

## Abstract

A controversy about the Swedish strategy of dealing with COVID-19 during the early period is how decision-making was based on evidence, which refers to data and data analysis. During the earliest period of the pandemic, the Swedish decision-making was based on subjective perspective. However, when more data became available, the decision-making stood on mathematical and descriptive analyses. The mathematical analysis aimed to model the condition for herd immunity while the descriptive analysis compared different measures without adjustment of population differences and updating pandemic situations. Due to the dubious interpretations of these analyses, a mild measure was adopted in Sweden upon the arrival of the second wave, leading to a surge of poor public health outcomes compared to the other Nordic countries (Denmark, Norway, and Finland). In this article, using data available during the first wave, we conduct longitudinal analysis to investigate the consequence of the shred of evidence in the Swedish decision-making for the first wave, where the study period is between January 2020 and August 2020. The design is longitudinal observational study. The linear regressions based on the Poisson distribution and the binomial distribution are employed for the analysis. We found that the early Swedish measure had a long-term and significant effect on general mortality and COVID-19 mortality and a certain mitigating effect on unemployment in Sweden during the first wave; here, the effect was measured by an increase of general deaths, COVID-19 deaths or unemployed persons under Swedish measure relative to the measures adopted by the other Nordic countries. These pieces of statistical evidence were not studied in the mathematical and descriptive analyses but could play an important role in the decision-making at the second wave. In conclusion, a timely longitudinal analysis should be part of the decision-making process for containing the current pandemic or a future one.

## Highlights

• Different measures led to different public health and economic outcomes of COVID-19.
• Decision-making of these measures was based on evidences, but
• Which evidences were used during the dramatic first wave of COVID-19?

## 1 Introduction

Since the World Health Organization declared the coronavirus disease 2019 (COVID-19) as a pandemic on 11 March 2020, countries around the globe have adopted different strategies of combating the transmission of COVID-19 while alleviating its negative impact on public health and the economy. Sweden was representative of those strategies, emphasizing the mitigation of transmission and taking stepwise mild measures (Erica, 2021; Kavaliunas, Ocaya, Mumper, Lindfeldt, & Kyhlstedt, 2020; Ludvigsson, 2020). On the other hand, the other Nordic countries, i.e., Denmark, Finland, and Norway, were representative of the common strategies, emphasizing the suppression of transmission and taking invasive measures (Erica, 2021; Lindström, 2020, 2021). During the early period of the pandemic, the two strategies led to obvious different results; for instance, the COVID-19 mortality per 100,000 individuals in Sweden versus the other Nordic countries is 58.15 versus 7.34 between March 2020 and August 2020 and 69.71 versus 13.40 between September 2020 and February 2021. The two periods represent two waves of the pandemic progression in the Nordic countries, each completing a cycle of rising, plateau, and decline and base for public health outcomes such as COVID-19 deaths.

A major controversy about the Swedish strategy of dealing with COVID-19 concerns how the decision-making was made. As repeatedly stated by the Swedish public health agency, the de facto administrator for handling the pandemic, the decision-making was based on evidence (Irwin, 2020; Lindström, 2020, 2021; Sayers 2020); see also CNN interview on 17 April with the former state epidemiologist Johan Giesecke (1995–2005) (CNN, 2020). Notably, this statement is in line with the general framework of Epidemiology and Evidence-Based Medicine. Here, evidence refers to data and data analysis; data is observational as typical of a pandemic; data analysis compares different measures for public health and economic outcomes, and it can be mathematical, descriptive, or statistical. In addition, there are discussions in the literature on the influence of social factors on decision-making (Erica, 2021; Irwin, 2020; Kavaliunas et al., 2020; Lindström, 2020, 2021; Ludvigsson, 2020). The major social factors are the emphasis on individual freedom and choice, the stress on acquiring knowledge by self-learning in the Swedish culture, and the top-down consensus culture and the structure of media policy. Although these social factors impact decision-making, they do not directly affect public health and economic outcomes. Therefore, they are irrelevance to evidence and not included in the data analysis.

During the very initial period of COVID-19, little data was available. Therefore, based on the subjective perspective, a mild measure was adopted in Sweden (Erica, 2021; Kavaliunas et al., 2020; Lindström, 2020; Ludvigsson, 2020). As the pandemic progressed, data became available, often in the form of tables. Given data, the decision-making was based on mathematical analyses and descriptive analyses. The mathematical analysis only modeled the condition for herd immunity (Britton, Ball, & Trapman, 2020). Nevertheless, the mathematical analysis had a considerable impact on the decision-making of the Swedish strategy during the first and second waves of this pandemic (Irwin, 2020; Lindström, 2020, 2021; Sayers, 2020). Descriptive analyses compared different measures of dealing with COVID-19 for public health and economic outcomes. However, they were only cross-sectional and without adjustment for population differences and updating pandemic situations.

Based on descriptive analyses, a group of Swedish scientists (i.e., the Science Forum COVID-19) called for stricter measures like those in the other Nordic countries (Bjermeret al., 2020), together with WHO and others (BBC, 2020; Claeson & Hanson, 2020; Claeson & Hanson, 2021; Habib, 2020; Roxby & Gure, 2020; Vogel, 2020). Also, based on descriptive analyses, the curve gradually flattened of COVID-19 incidences and hospitalizations and COVID-19 and general deaths since May 2020 (Erica, 2021). The general mortality even fell lower in Sweden than in the other Nordic countries. Due to the dubious interpretations of the descriptive and mathematical analyses, the Swedish public health agency continued with the mild measure recommendations during the second wave. However, this led to a surge of poor public health outcomes compared to the other Nordic countries (Erica, 2021), see also the weekly reports from the public health agencies of the Nordic countries.

On the other hand, pandemic progression is a complex stochastic process in which measures yield outcomes and outcomes in turn influence subsequent measures. In this context, the measure taken during a period has not only a short-term effect on the immediate outcome in the same period but also a long-term effect on the outcomes in subsequent periods. In the decision-making, statistical analysis is essential, which can be cross-sectional and longitudinal and allows for adjustment of population differences and updating pandemic situations. To the best of our knowledge, most of the current statistical analyses in the literature only address the short-term influences of various measures on the outcome (Brauner, 2021; Flaxman et al., 2020; Haug et al., 2020; Kontis, 2020; Soltesz et al., 2020).

One can ask if there was any evidence being missed in the Swedish decision-making? What were the consequences of evidence missing? In this article, we conduct a longitudinal observational study to examine the causal effect of the Swedish strategy relative to the common strategy adopted by the other Nordic countries on public health and economic outcomes during the first wave of the pandemic. We compare our analytical results to descriptive analysis to investigate what was missed for the decision-making of the Swedish strategy of dealing with the second wave. Furthermore, using the same table data as with descriptive analyses, we demonstrate that statistical evidence can be provided in time for the decision makers.

## 2 Material and the premises for statistical analyses

### 2.1 Data sources

All data are publicly available. The COVID-19 mortality and general mortality are obtained from the Swedish National Board of Health and Welfare (https://www.government.se/government-agencies/national-board-of-health-and-welfare--socialstyrelsen/), Statistics Denmark (https://www.dst.dk/en), the Norwegian Institute of Public Health (https://www.fhi.no/en/), the Finnish Institute for Health and Welfare (https://thl.fi/en/web/thlfi-en) and Statistics Finland (https://www.stat.fi/index_en.html). As recommended by the WTO, all four Nordic countries identified COVID-19 death as death for which a positive COVID-19 PCR test was recorded within the 30 days.

Unemployment was measured as the number of unemployed persons aged 15–74, and the employment as the number of employed persons aged 15–74. These numbers were produced by the labor force surveys conducted in individual countries following the European Union Council Regulation. The labor force was the sum of employed and unemployed persons. Population density was measured as the number of inhabitants per square kilometers. These numbers are obtained from Statistics Sweden (https://www.scb.se/en), Statistics Denmark (https://www.dst.dk/en), Statistics Norway (https://www.ssb.no/en), and Statistics Finland (https://www.stat.fi/index_en.html).

### 2.2 Pandemic progression with public health outcomes and the confounding adjustment

For the sake of explication, we consider public health outcomes here. Economic outcomes will be similarly studied in Section 3.5.

The public health outcomes refer to COVID-19 incidence, admission to hospitalization and intensive care, general death, and COVID-19 death. However, the Nordic countries had different policies for admission to hospitals and intensive care. For instance, Denmark had a much higher admission rate than Sweden (Erica, 2021; Kavaliunas et al., 2020; Lindström, 2020; Ludvigsson, 2020). Similarly, groups of tested people were hardly random samples from the population. Therefore, it would be problematic to analyze the admission to hospitalization and intensive care and the testing of COVID-19. On the other hand, reported deaths were far more reliable than incidence-related data; for instance, only a small number of early deaths attributable to COVID-19 might have been missed. Therefore, we use general death and COVID-19 death in this article as our public health outcomes.

The initial period of the pandemic took place around weeks 10–18 in the Nordic countries; please note that week 1, 2020 corresponds to the dates from 30 December 2019 to 5 January 2020. Weeks 10–35 completed a cycle of rising, plateau, and decline and base for the public health outcome, and are considered the first wave of the pandemic (Erica, 2021; Ludvigsson, 2020). Because it is impossible to know when measures became effective, we divide the entire follow-up into four periods of approximately equal length: weeks 1–9, 10–18, 19–26, and 27–35. Let period t (t=1,2,3) indicate the three periods during weeks 10–35: period 1 for weeks 10–18, period 2 for weeks 19–26, and period 3 for weeks 27–35. In Supplementary Materials, we conduct a sensitivity analysis to show that when alternatively dividing the entire follow-up into weeks 1–9, 10–17, 18–26, 27–35, the result only differs slightly, and the conclusion is the same.

During weeks 1–9, the pandemic had not yet broken out, so no measure was adopted, and there was only the outcome $y_{0}$ for general mortality. During period 1, the exposure was $z_{1}=1$ for the Swedish measure or 0 for the common measure and yielded outcome $y_{1}$. From here and on, the common measures refer to those adopted by the other Nordic countries. During period 2, the exposure was $z_{2}=1$ for the Swedish measure or 0 for the common measure and yielded outcome $y_{2}$. During period 3, the exposure was $z_{3}=1$ for the Swedish measure or 0 for the common measure and yielded outcome $y_{3}$. Here the outcomes $y_{1}\text{,}$ $y_{2}$ and $y_{3}$ were general mortalities or COVID-19 mortalities.

Outcome $y_{0}$ represents the initial health status and has influences on outcomes $y_{1}$, $y_{2}$ and $y_{3}$. Thus, it is a stationary covariate and may confound the causal effects of exposures $z_{1}$, $z_{2}$ and $z_{3}$. Outcome $y_{1}$ represents the updating pandemic situation under exposure $z_{1}$ and has influences on outcomes $y_{2}$ and $y_{3}$. Thus, it is also a covariate and may confound the causal effects of exposures $z_{2}$ and $z_{3}$. Outcome $y_{2}$ represents the updating pandemic situation under exposure $z_{2}$ and has influences on outcome $y_{3}$. Thus, it is also a covariate and may confound the causal effect of exposure $z_{3}$.

Besides initial general mortality $y_{0}$, there might exist other stationary covariates that characterize populations in the Nordic countries. However, these countries are similar to one another in terms of economy, culture, and society, so most of the stationary covariates, such as gender, education, and socioeconomic status, have similar distributions among these countries and thus do not confound the causal effects of exposures $z_{1}$, $z_{2}$ and $z_{3}$. As a result, there is no need to adjust for these covariates, as is a common practice in statistical analyses. Table 1 lists some characteristics of the populations in the Nordic countries. As seen in this table, the initial general mortality $y_{0}$ and population density x differs considerably in different regions of these countries and may confound the causal effects of these exposures. Therefore, we divide Sweden into six regions: Stockholm, Skåne, Gothenburg, Halland, Västmanland, and the rest of Sweden. Because COVID-19 mortality was low in Norway, Denmark, and Finland, we do not divide these countries into small regions. The stationary covariates, exposures, outcomes, and follow-ups are summarized in Table 2.

**Table 1:** Characteristics of study populations in regimes of the Nordic countries before the breakout of COVID-19: (1) Stockholm, (2) Skåne, (3) Göteborg, (4) Halland, (5) Västmanland, (6) the rest of Sweden, (1–6) Sweden as a whole, (7) Denmark, (8) Norway, (9) Finland.

	Populations in regions									
Characteristics	(1)	(2)	(3)	(4)	(5)	(6)	(1–6)	(7)	(8)	(9)
Population size^a^, ${n\;10}^{3}$	2377	1378	1726	334	276	4237	10328	5828	5328.2	5525.3
Sex^a^, $n\;10^{3}$ (%)										
Male	1190(50.06)	688(49.93)	870(50.41)	168(50.30)	139(50.36)	2142(50.54)	5196(50.31)	2899(49.75)	2685(50.39)	2728(49.38)
Female	1187(49.94)	690(50.07)	856(49.59)	166(49.70)	137(49.64)	2096(49.46)	5132(49.69)	2928(50.25)	2643(49.61)	2797(50.62)
Age group^a^, $n\;10^{3}$(%)										
0–19 years,	571 (24.03)	327(23.73)	398(23.06)	80 (23.95)	64 (23.19)	964 (22.75)	2404(23.28)	1298(22.28)	1255(23.55)	1168(21.14)
20–64 years	1426(60.02)	781(56.68)	992(57.47)	181(54.19)	152(55.07)	2326(54.90)	5858(56.72)	3377(57.95)	3154(59.20)	3126(56.58)
65- years	379 (15.95)	270(19.59)	336(19.47)	73 (21.86)	60 (21.74)	947 (22.35)	2065(20.00)	1152(19.77)	919 (17.25)	1231(22.28)
Population density^a^, n per ${km}^{2}$	365	128	73	62	54	11	25	137	15	18
General mortality rate^b^, n per 100,000 person weeks	13.8	17.6	18.0	17.5	19.5	20.4	18.0	18.4	15.9	19.4
Unemployment rate^c^, %	7.0	11.1	7.0	6.3	9.0	8.6	7,6	5.4	3.8	7.7

Over a long period of time, all four countries are similar in terms of social and economic systems, social welfare systems including public health policies, education systems, and cultural traditions.

Due to slightly different categorization of these social characteristics among these countries, their statistics are not listed here. Interested readers are referred to official statistics available on the webpages of Statistics Sweden, Statistics Denmark, Statistics Norway, and Statistics Finland.

a Based on December 2019.

b Based on weeks 1–9, 2020

c Based on quarter 1, 2020.

**Table 2:** A summary of population density, exposures, outcomes, and the follow-ups during different periods. The outcome can also be a covariate for the subsequent exposures. The study was conducted between August 2021 and October 2021. The geographical location of the study population was the Nordic countries (Sweden, Denmark, Norway, and Finland). The study population was those of the Nordic countries, and the demographics are given in Table 1.

Period	Population density (persons per ${\text{km}}^{2}$)	Exposure: 1 for the Swedish measure 0 for the common measure	Outcome: Covid-19 mortality or general mortality	Follow-up (Person weeks)
Weeks 1–9	x	none	$y_{0}$	$p_{0}$
Weeks 10–18 (Period 1)	x	$z_{1}=1$ or $z_{1}=0$	$y_{1}$	$p_{1}$
Weeks 19–26 (Period 2)	x	$z_{2}=1$ or $z_{2}=0$	$y_{2}$	$p_{2}$
Weeks 27–35 (Period 3)	x	$z_{3}=1$ or $z_{3}=0$	$y_{3}$	$p_{3}$

### 2.3 Assumption of no hidden confounding covariates and effect measure

To summarize the confounding situation in the pandemic, we assume no hidden confounding covariates: (a) conditional on population density x and outcome $y_{0}$, no other covariates confound the causal effect of the exposure sequence $\left(z_{1},\;z_{2},z_{3}\right)$; (b) conditional on population density x and outcome $y_{1}$, no other covariates confound the causal effect of the exposure sequence $\left(z_{2},z_{3}\right)$; (c) conditional on population density x and outcome $y_{2}$, no other covariates confound the causal effect of exposure $z_{3}$. The assumption implies that to study the causal effects of exposures, we need to compare the outcomes of the exposures on the same level of population density and the most recent outcome before these exposures. With the assumption and data, we will estimate causal effects during the pandemic progression in Section 3 in the framework of sequential causal inference (Hernan & Robins, 2020; Wang & Yin, 2020). All causal effects will be measured by an increase of outcome $y_{1}$, $y_{2}$ or $y_{3}$ under Swedish measure relative to the measures adopted by the other Nordic countries (common measure).

## 3 Statistical analyses and the results

### 3.1 Analytic strategy

We will estimate three types of causal effects of Swedish measures relative to the common measures: short-term causal effects, sequential causal effects, and long-term causal effects. The short-term causal effect compares Swedish measure $z_{t}=1$ versus common measure 0 for the immediate outcome $y_{t}$. The sequential causal effect compares Swedish sequence versus common sequence for a remote outcome, for instance, Swedish sequence $\left(z_{1},\;z_{2},z_{3}\right)=\left(1,\;1,\;1\right)$ versus common sequence (0,0,0) for the remote outcome $y_{3}$. The exposures/exposure sequences are observed for these causal effects, so we can apply regressions to estimate them, that is, the Poisson distribution-based linear regression for general mortality and COVID-19 mortality as count numbers in follow-ups of the population and the binomial distribution-based linear regression for unemployment as a count number from a sample of the population.

The long-term causal effect compares, for instance, mixed sequence $\left(z_{1},\;z_{2},z_{3}\right)=\left(1,\;0,\;0\right)$ to common sequence (0,0,0) for the remote outcome $y_{3}$. Because mixed sequence cannot be observed, we cannot apply regression to estimate the long-term causal effect. Due to Robins (Hernan & Robins, 2020), sequential causal inference is developed to estimate long-term causal effects under unobserved sequences of exposures by using observed data. Notably, the new general formula (G-formula) reveals a rather intuitive observation that the causal effect of a sequence of exposures must be a sum of contributions of individual exposures in the sequence (Wang & Yin, 2020). The new G-formula allows us to estimate the long-term causal effect from the estimated short-term causal effect and the estimated sequential causal effect without introducing additional modeling assumptions. In the following subsections, we will describe statistical analyses and the results in detail.

### 3.2 Short-term causal effects of the Swedish measures on public health outcomes

There are three short-term causal effects (1), (2) and (3), as described and estimated below. To be precise, causal effect (1) is an increase in outcome $y_{1}$ under the Swedish measure $z_{1}=1$ relative to the common measure 0 during period 1. Causal effect (2) is an increase in outcome $y_{2}$ under the Swedish measure $z_{2}=1$ relative to the common measure 0 during period 2. Causal effect (3) is an increase in outcome $y_{3}$ under the Swedish measure $z_{3}=1$ relative to the common measure 0 during period 3.

Because the outcomes are observed under both the Swedish and common measures, we conduct regression to estimate causal effects (1)–(3), as described in Appendix; the Poisson distribution-based linear regression yields the estimates, 95% confidence intervals (CIs), and p-values for these causal effects. The estimation of short-term causal effects or their trend is often seen in the time series and panel data analyses. In Appendix, we describe these regressions for causal effects (1)–(3) in detail. We also conduct descriptive analysis, in which the difference in means estimates the causal effect.

Table 3 presents the results of causal effects (1)–(3) from both statistical analysis and descriptive analysis. As shown by statistical analysis (columns 2 and 4 of Table 3), the Swedish strategy continually improved its performance in terms of general mortality and COVID-19 mortality along weeks 10–18, 19–26, and 27–35 (periods 1, 2, and 3) and performed even better in terms of the general mortality during weeks 19–26 and 27–35 (periods 2 and 3), i.e., flattened curve (Erica, 2021; Kavaliunas et al., 2020; Ludvigsson, 2020). Although descriptive analysis (columns 3 and 5) yields the same trend for causal effects (1)–(3) as statistical analysis (columns 2 and 4), it is not adjusted for confounding. Consequently, its interpretation is dubious.

**Table 3:** The short-term causal effect of the Swedish measure relative to the common measure adopted by the other Nordic countries on public health outcome: estimate, 95% confidence interval, and the p-value. The descriptive analysis yields only crude estimates. All causal effects are measured per 100,000 individuals. The study was conducted between August 2021 and October 2021. The geographical location of the study population was the Nordic countries (Sweden, Denmark, Norway, and Finland). The study population was those of the Nordic countries, and the demographics are given in Table 1. Causal effect (1): An increase in outcome $y_{1}$ under the Swedish measure $z_{1}=1$ relative to the common measure $z_{1}=0$ during period 1 (weeks 10–18). Causal effect (2): An increase in outcome $y_{2}$ under the Swedish measure $z_{2}=1$ relative to the common measure $z_{2}=0$ during period 2 (weeks 19–26). Causal effect (3): An increase in outcome $y_{3}$ under the Swedish measure $z_{3}=1$ relative to the common measure $z_{3}=0$ during period 3 (weeks 27–35).

Causal effect	$\left(\begin{array}{c} Estimate\\ 95\%\;CI\\ p-value \end{array}\right)$ for the short-term causal effect on public health			
	General mortality		COVID-19 mortality	
	Statistical analysis	Descriptive analysis	Statistical analysis	Descriptive analysis
(1)	20.2	29.0	18.7	25.4
	(16.6, 23.7)		(17.6, 19.8)	
	<0.001		<0.001	
(2)	−2.2	14.9	14.4	21.2
	(−7.4, 3.1)		(12.8, 16.0)	
	<0.4188		<0.001	
(3)	−17.6	−5.7	1.9	3.1
	(−22.5, −12.6)		(0.5, 3.3)	
	<0.001		<0.007	

### 3.3 Sequential causal effects of the Swedish sequences on public health outcomes

There are three sequential causal effects (4), (5), and (6), as described and estimated below. To be precise, causal effect (4) is an increase in outcome $y_{2}$ during period 2 under the Swedish sequence $\left(z_{1},\;z_{2}\right)=\left(1,\;1\right)$ relative to the common sequence (0,0) during periods 1 and 2. Causal effect (5) is an increase in outcome $y_{3}$ during period 3 under the Swedish sequence $\left(z_{2},\;z_{3}\right)=\left(1,\;1\right)$ relative to the common sequence (0,0) during periods 2 and 3. Causal effect (6) is an increase in outcome $y_{3}$ during period 3 under the Swedish sequence $\left(z_{1},\;z_{2},\;z_{3}\right)=\left(1,\;1,\;1\right)$ relative to the common sequence (0,0,0) during periods 1, 2 and 3.

Usually, it is difficult to estimate the sequential causal effect because the outcomes are not observed under exposure sequences. However, in the context of the pandemic, the outcomes are observed under both the Swedish and common sequences, so causal effects (4)–(6) can be estimated by the regression, i.e., the Poisson distribution-based linear regression, which, as described in Appendix, yields the estimates, 95% CIs and p-values for these causal effects. Noticeably, it is impossible to estimate causal effects (4)–(6) by descriptive analysis. In Appendix, we describe these regressions for causal effects (4)–(6) in detail. Table 4 presents the results of causal effects (4)–(6) from statistical analysis.

**Table 4:** The sequential causal effect of the Swedish sequence relative to the common sequence adopted by the other Nordic countries on public health outcome: estimate, 95% confidence interval, and the p-value. All causal effects are measured per 100,000 individuals. The study was conducted between August 2021 and October 2021. The geographical location of the study population was the Nordic countries (Sweden, Denmark, Norway, and Finland). The study population was those of the Nordic countries, and the demographics are given in Table 1. Causal effect (4): An increase in outcome $y_{2}$ during period 2 (weeks 19–26) under the Swedish sequence $\left(z_{1},\;z_{2}\right)=\left(1,\;1\right)$ relative to the common sequence (0,0) during periods 1 and 2 (weeks 10–26). Causal effect (5): An increase in outcome $y_{3}$ during period 3 (weeks 27–35) under the Swedish sequence $\left(z_{2},\;z_{3}\right)=\left(1,\;1\right)$ relative to the common sequence (0,0) during periods 2 and 3 (weeks 19–35). Causal effect (6): An increase in outcome $y_{3}$ during period 3 (weeks 27–35) under the Swedish sequence $\left(z_{1},\;z_{2},\;z_{3}\right)=\left(1,\;1,\;1\right)$ relative to the common sequence (0,0,0) during periods 1, 2, and 3 (weeks 10–35).

Causal effect	$\left(\begin{array}{c} Estimate\\ 95\%\;CI\\ p-value \end{array}\right)$ for the sequential causal effect on public health	
	General mortality	COVID-19 mortality
(4)	11.9	20.3
	(8.6, 15.2)	(19.3, 21.2)
	<0.001	<0.001
(5)	−17.4	3.3
	(−22.1, −12.6)	(2.7, 4.0)
	<0.001	<0.001
(6)	−7.3	3.15
	(−10.6, −4.0)	(2.8, 3.5)
	<0.001	<0.001

Causal effects (4)–(6) are of little interest by themselves. However, we use them to obtain the following long-term causal effects of the Swedish measures.

### 3.4 Long-term causal effects of the Swedish measures on public health outcomes

There are three long-term causal effects (7), (8), and (9), as described and estimated below. To be precise, causal effect (7) is an increase in outcome $y_{2}$ during period 2 under the mixed sequence $\left(z_{1},\;z_{2}\right)=\left(1,\;0\right)$ relative to the common sequence (0,0) during periods 1 and 2. It describes the long-term influence of the Swedish measure during period 1 on the outcome during period 2. The mixed sequence and its outcome have never been observed, so the regression cannot estimate causal effect (7). However, according to formula (18) in Theorem 2 of Wang and Yin (Wang & Yin, 2020), we have the following relationship between causal effects

causaleffect(4)=causaleffect(7)+causaleffect(2).

This formula is an example of the new G-formula (Wang & Yin, 2020), which describes a rather intuitive observation that the causal effect of a sequence of exposures must be a sum of contributions of individual exposures in the sequence. In Supplementary Materials, we describe this equality in more detail. Notably, the relationship between causal effects (4), (7), and (2) is implied by their exposure sequences $\left(z_{1},z_{2}\right)=\left(1,\;1\right)$, $\left(z_{1},z_{2}\right)=\left(1,\;0\right)$, and $z_{2}=1$. By applying this equality to causal effects (4) and (2) estimated from Section 3.2, 3.3, we obtain the estimate, 95% CI, and p-value for causal effect (7).

Causal effect (8) is an increase in outcome $y_{3}$ during period 3 under the mixed sequence $\left(z_{2},\;z_{3}\right)=\left(1,\;0\right)$ relative to the common sequence (0,0) during periods 2 and 3, and it describes the long-term influence of the Swedish measure during period 2 on the outcome during period 3. According to formula (18) in Theorem 2 of Wang and Yin (Wang & Yin, 2020) and as described in Supplementary Materials, we have the following relationship between causal effects

causaleffect(5)=causaleffect(8)+causaleffect(3).

Please note that the exposure sequences in causal effects (5), (8), and (3) are$\left(z_{2},\;z_{3}\right)=\left(1,\;1\right)$, $\left(z_{2},\;z_{3}\right)=\left(1,\;0\right)$, and $z_{3}=1$. By applying this equality to causal effects (5) and (3) estimated from Section 3.2, 3.3, we obtain the estimate, 95% CI and p-value for causal effect (8).

Causal effect (9) is an increase in $y_{3}$ during period 3 under the mixed sequence $\left(z_{1},z_{2},\;z_{3}\right)=\left(1,\;0,\;0\right)$ relative to the common sequence (0,0,0) during periods 1, 2 and 3, and it describes the long-term influence of the Swedish measure during period 1 on the outcome during period 3. According to formula (18) in Theorem 2 of Wang and Yin (Wang & Yin, 2020) and as described in Supplementary Materials, we have the following relationship between causal effects

causaleffect(6)=causaleffect(9)+causaleffect(8)+causaleffect(3).

Please note that the exposure sequences in causal effects (6), (9), (8), and (3) are$\left(z_{1},\;z_{2},\;z_{3}\right)=\left(1,\;1,\;1\right)$, $\left(z_{1},\;z_{2},\;z_{3}\right)=\left(1,\;0,\;0\right)$, $\left(z_{2},\;z_{3}\right)=\left(1,\;0\right)$ and $z_{3}=1$. By applying this equality to causal effects (6), (8) and (3) estimated earlier, we obtain the estimate, 95% CI and p-value for causal effect (9).

Table 5 presents the results of causal effects (7)–(9) from statistical analysis. As shown in Table 5, the early Swedish measure during weeks 10–18 (period 1) had long-term causal effects (7) and (9): it led, per 100,000 individuals, to 14.0 (95% CI 10.2–17.9) more general deaths during weeks 19–26 (period 2) and 10.1 (6.6, 13.6) more general deaths during weeks 27–35 (period 3) as well as 5.9 (4.4, 7.3) more COVID-19 deaths during weeks 19–26 (period 2).

**Table 5:** The long-term causal effect of the Swedish measure relative to the common measure adopted by the other Nordic countries on public health outcome: estimate, 95% confidence interval, and the p-value. All causal effects are measured per 100,000 individuals. The study was conducted between August 2021 and October 2021. The geographical location of the study population was the Nordic countries (Sweden, Denmark, Norway, and Finland). The study population was those of the Nordic countries, and the demographics are given in Table 1. Causal effect (7): An increase in outcome $y_{2}$ during period 2 (weeks 19–26) under the mixed sequence $\left(z_{1},z_{2}\right)=\left(1,\;0\right)$ relative to the common sequence (0,0) during periods 1 and 2 (weeks 10–26). Causal effect (8): An increase in outcome $y_{3}$ during period 3 (weeks 27–35) under the mixed sequence $\left(z_{2},z_{3}\right)=\left(1,\;0\right)$ relative to the common sequence (0,0) during periods 2 and 3 (weeks 19–35). Causal effect (9): An increase in outcome $y_{3}$ during period 3 (weeks 27–35) under the mixed sequence $\left(z_{1},\;z_{2},z_{3}\right)=\left(1,\;0,\;0\right)$ relative to the common sequence (0,0,0) during periods 1, 2, and 3 (weeks 10–35).

Causal effect	$\left(\begin{array}{c} Estimate\\ 95\%\;CI\\ p-value \end{array}\right)$ for the long-term causal effect on public health	
	General mortality	COVID-19 mortality
(7)	14.0	5.9
	(10.2, 17.9)	(4.4, 7.3)
	<0.001	<0.001
(8)	0.2	1.4
	(−4.9, 5.2)	(0.2, 2.7)
	0.947	0.024
(9)	10.1	−0.2
	(6.6, 13.6)	(−0.8, 0.4)
	0.001	0.505

### 3.5 Causal effects of the Swedish strategy on unemployment

Here, we study the outcome of unemployment in an analogy to public health outcomes. We divide the first nine months of 2020 into quarters 1, 2, and 3. During quarter 1, measures were not adopted, and even if some measures had been taken, they should not have influenced unemployment in the current quarter, so there was only unemployment $y_{1}$ from labor force $p_{1}$ in quarter 1. During quarter 2, the exposure was $z_{2}=1$ for the Swedish measure or 0 for the common measure, yielding an unemployment $y_{2}$ in labor force $p_{2}$. During quarter 3, the exposure was $z_{3}=1$ for the Swedish measure or 0 for the common measure, yielding an unemployment $y_{3}$ in labor force $p_{3}$. Here, unemployment refers to the number of unemployed persons aged 15–74, and labor force to the number of both unemployed and employed persons aged 15–74.

To adjust for confounding, we have the following assumption of no hidden confounding covariates: (a) conditional on population density x and outcome $y_{1}$, no other covariates confound the causal effect of a sequence $\left(z_{2},z_{3}\right)$ of exposures; (b) conditional on population density x and outcome $y_{2}$, no other covariates confound the causal effect of exposure $z_{3}$. With the assumption and data, we will estimate four causal effects during the pandemic progression in the framework of sequential causal inference (Hernan & Robins, 2020; Wang & Yin, 2020). All causal effects are measured by an increase of outcome $y_{2}$, or $y_{3}$ under the Swedish measure relative to the common measure adopted by the other Nordic countries.

We first estimate the following three causal effects: (1) an increase in unemployment $y_{2}$ under the Swedish measure $z_{2}=1$ relative to the comment measure 0 during quarter 2; (2) an increase in unemployment $y_{3}$ under the Swedish measure $z_{3}=1$ relative to the common measure 0 during quarter 3; (3) an increase in outcome $y_{3}$ during quarter 3 under the Swedish sequence $\left(z_{2},\;z_{3}\right)=\left(1,\;1\right)$ relative to the common sequence (0,0) during quarters 2 and 3. Causal effects (1)–(2) are the short-term causal effects of the Swedish measures on unemployment. Causal effect (3) is the sequential causal effect of the Swedish sequence on unemployment. Because unemployment is observed under both the Swedish measure or sequence and the common measure or sequence, we may conduct regression, i.e., the binomial distribution-based linear regression, which, as described in Appendix, yields the estimates, 95% CIs and p-values for causal effects (1)–(3).

Now we estimate causal effect (4), which is an increase in unemployment $y_{3}$ during quarter 3 under the mixed sequence $\left(z_{2},z_{3}\right)=\left(1,\;0\right)$ relative to the comment sequence (0,0) during quarters 2 and 3. It is the long-term causal effect of the Swedish measure during quarter 2 on unemployment during quarter 3. By the same argument as with the public health outcome, we have

causaleffect(3)=causaleffect(4)+causaleffect(2).

Please note that the exposure sequences in causal effects (3), (4), and (2) are $\left(z_{2},z_{3}\right)=\left(1,\;1\right)$, $\left(z_{2},z_{3}\right)=\left(1,\;0\right)$ and $z_{3}=1$. By applying this equality to causal effects (3) and (2) estimated earlier, we obtain the estimate, 95% CI and p-value for causal effect (4).

We also conduct a descriptive analysis, in which the difference in means estimates causal effects (1) and (2). Table 6 presents the results of causal effects (1)–(4) from both statistical analysis and descriptive analysis.

**Table 6:** The causal effect of the Swedish strategy relative to the common strategy adopted by the other Nordic countries on unemployment: estimate, 95% confidence interval, and the p-value. The descriptive analysis yields only crude estimates for causal effects (1) and (2). All causal effects are measured per 100,000 individuals. The study was conducted between August 2021 and October 2021. The geographic location of the study population was the Nordic countries (Sweden, Denmark, Norway, and Finland). The study population was those of the Nordic countries, and the demographics are given in Table 1. Causal effect (1): An increase in unemployment $y_{2}$ under the Swedish measure $z_{2}=1$ relative to the comment measure $z_{2}=0$ during quarter 2. Causal effect (2): An increase in unemployment $y_{3}$ under the Swedish measure $z_{3}=1$ relative to the comment measure $z_{3}=0$ during quarter 3. Causal effect (3): An increase in unemployment $y_{3}$ during quarter 3 under the Swedish sequence $\left(z_{2},\;z_{3}\right)=\left(1,\;1\right)$ relative to the common sequence (0,0) during quarters 2 and 3. Causal effect (4): An increase in unemployment $y_{3}$ during quarter 3 under the mixed sequence $\left(z_{2},z_{3}\right)=$(1,0) relative to the common sequence (0,0) during quarters 2 and 3. Causal effects (1) and (2) are the short-term causal effects of the Swedish measures on unemployment. Causal effect (3) is the sequential causal effect of the Swedish sequence on unemployment. Causal effect (4) is the long-term causal effect of the Swedish measure on unemployment.

Causal effect	$\left(\begin{array}{c} Estimate\\ 95\%\;CI\\ p-value \end{array}\right)$ for the causal effect on unemployment	
	Statistical analysis	Descriptive analysis
(1)	585.0	2944.1
	(523.9, 646.0)	
	<0.001	
(2)	528.4	2226.6
	(480.2, 576.5)	
	<0.001	
(3)	624.5	
	(576.9, 672.1)	
	0.01	
(4)	96.1	
	(59.0, 133.3)	
	<0.001	

The statistical analysis (column 2 of Table 6) reveals the following. First, as shown from causal effects (1) and (2), the Swedish strategy performed worse than the common strategy in terms of unemployment. Second, as demonstrated from causal effect (4), the Swedish measure during quarter 2 had a certain long-term mitigating influence on unemployment: it led to, per 100,000 individuals, only 96.1 (59.0, 133.3) more unemployment during quarter 3.

With the descriptive analysis (column 3 of Table 6), causal effects (4) and (5) are not estimable. Therefore it is impossible to assess the long-term mitigating influence of the early Swedish measure on unemployment. Furthermore, as shown from causal effects (1) and (2) in column 3, the crude estimates obtained from the descriptive analysis are far more significant than those obtained from statistical analysis, possibly due to the fact that the initial unemployment rate differs considerably in these countries and confounds the causal effect. Therefore it is difficult to interpret these crude estimates.

## 4 Conclusions and discussions

This article conducts an observation longitudinal analysis for the first wave of the pandemic progression and has two major findings. First, the early mild measure had a long-term and significant influence on general mortality and COVID-19 mortality. Second, the early mild measure led to a certain degree of long-term mitigating effect on unemployment.

The analysis in this article contributes to the literature in two aspects of evaluating public policies. First, to the best of our knowledge, the long-term causal effect of public policy is not sufficiently studied in the evaluation of public policies. Our analysis demonstrates that the long-term causal effect of public policy can be estimated in the framework of sequential causal inference (Hernan & Robins, 2020). Second, the data used for our analysis is the same table data as used for descriptive analyses. As demonstrated by our analysis, statistical analysis can be conducted at the same time as descriptive analysis. Statistical evidence from our analysis could have impacted the decision-making for the second wave of the pandemic in Sweden.

Our statistical analysis may provide insight into two major paradoxes in the decision-making of the Swedish strategy. One major paradox concerns which evidence was used (Lindström, 2020, 2021). During the initial period of the pandemic, it was not realistic to design and conduct randomized trials for testing the influences of various measures, e.g., wearing a face mask. Similarly, it was difficult to design and conduct statistical analyses based on individual-level data. However, statistical analyses based on available table data were not conducted either to provide statistical evidence during the first and second waves, so the policy maker needed to rely on descriptive analyses. On the other hand, the evidence from descriptive analyses was dubious and of low level. Descriptive analyses might have missed crucial evidence such as the long term influence of the early Swedish measure, leading to the mild measure upon the arrival of the second wave. Our statistical analyses based on table data might have provided medium-level evidence for the decision-making in place of randomized trials or statistical analyses based on individual-level data.

The other major paradox concerns the postmodern view of science in Swedish society (Lindström, 2020, 2021; Lundberg, 2020). In short, the postmodern view emphasizes the existence of multiple truths of a particular scientific problem and advocates the subjective perception of truth (Inglehart, 1997). In contrast, the modern view emphasizes only one truth and advocates the finding of the objective truth. Without adjustment for confounding, descriptive analyses yielded evidence with multiple interpretations, i.e. multiple truths or multiple decisions. This observation is in agreement with the postmodern view of multiple truths. With adjustment for confounding, statistical analyses yielded evidence closer to the truth, i.e., a decision better than others. This observation is the modern belief in one objective truth.

Notably, the two views of science agree on one point: it is impossible to find the truth, single or multiple. Our statistical analysis was built on the assumption of no hidden confounders. Although we could never prove the assumption, we could always assess its validity for some covariates by sensitivity analysis. As a compromise, the statistical analysis could provide essential aspects of the truth, which might have yielded a better decision.

Up until now, several waves of the pandemic have come and gone. As a result, statistical analyses including randomized trials have been conducted in various countries. Thanks to the evidence provided by these studies, the strategies adopted by various countries including Sweden become more effective. On the other hand, most of these statistical analyses were based on individual-level data and lagged far behind the progression of the pandemic; for instance, while statistical analyses focused on the effective period of the vaccine in combating the Delta variant of COVID-19, the pandemic already progressed to enter an era of the Omicron variant. Consequently, the current decision-making essentially stands on the current descriptive evidence and the old statistical evidence. Therefore, it is desirable to conduct statistical analyses with available data and provide timely statistical evidences for the decision makers before randomized trials and statistical analyses based on individual-level data become possible.

There are several limitations to our statistical analysis. The first limitation concerns the use of table data. Although statistical analysis based on table data may provide timely statistical evidence for the decision maker, the evidence is of low level compared to randomized trials and statistical analysis based on individual-level data. The second limitation concerns outcomes. The technical procedure of detecting and reporting COVID-19 death differed between these countries, particularly during the initial period of the pandemic, and this yielded certain biases in our analysis. The third limitation concerns exposures. In the current article, we broadly classified the strategies adopted by the other Nordic countries as the common strategy. This was clearly a simplifying approximation, which could help to focus on the Swedish strategy but omitted the differences with individual Nordic countries. The fourth limitation concerns covariates. Different countries might have different definitions for certain covariates, such as immigration status. Therefore it is not possible to adjust for immigration status in our analysis. It is less serious for our analysis because the Nordic countries have similar social-economic policies and cultures. However, it may become a serious problem if we compare Sweden with other countries.

## Author contribution

The three authors make equal contributions to the article.

## Funding

Partially supported by 10.13039/501100004359Swedish Research Council, Sweden with the grant numbers 2019-02913 and 2017-01175.

## Ethical approval

Not relevant.

## Data and code

Publicly available. Those producing the result are also given in Supplementary Materials.

## Declaration of competing interest

No conflict of interest.

## Appendix

### Estimating causal effects on public health outcomes

The outcomes $y_{1},\;y_{2},$and $y_{3}$ are general mortalities or COVID-19 mortalities. They are measured as count numbers in the population. Hence, we assume that the outcome follows the Poisson distribution conditional on the history of stationary covariates, exposures, and previous outcomes.

The population density x is measured by the number of inhabitants per square kilometers and it is a stationary covariate. During weeks 1–9, there was only general mortality $y_{0}$ and the amount $p_{0}$ of person weeks. Let $r_{1}=y_{0}/p_{0}$, which is the observed mortality rate before any measure was taken. As described in Section 2.2, let t=1,2,3 indicate three periods: t=1 for weeks 10–18, t=2 for weeks 19–26 and t=3 for weeks 27–35. Then during period t, we have exposure$z_{t}$ with $z_{t}=1$ for the Swedish measure and $z_{t}=0$ for the common measure, outcome $y_{t}$ for general mortality or COVID-19 mortality and the amount $p_{t}$ of person weeks. Furthermore, we have the observed mortality rate $r_{t}=y_{t-1}/p_{t-1}$ before measure $z_{t}$.

Causal effect (t) (t=1,2,3) is an increase in outcome $y_{t}$ under the Swedish measure $z_{t}=1$ relative to the common measure $z_{t}=0$ during period t. With the person weeks $p_{t}$, the regression model for the conditional expectation of $y_{t}$ given $\left(x,\;r_{t},\;z_{t}\right)$ is

$$E\left(y_{t}\;|\;x,\;r_{t},\;z_{t}\right)=p_{t}\left(\alpha_{t}+\gamma_{t}x+\delta_{t}r_{t}+\beta_{t}z_{t}\right),\;\text{for}\;t=1,\;2,\;3$$

Here, the link function is identity function; the outcome is mortality $y_{t}$ during period t; the covariates are density x and mortality rate $r_{t}$ during period t−1; the exposure is $z_{t}$ (Swedish or common measure); the amount $p_{t}$ of person weeks during period t is fixed as a constant. We use linear model with only the main effect of exposure $z_{t}$ for the following reasons. First, the exact functional form for the nuisance variables x and $r_{t}$ is unknown, and a reasonable assumption is linear form. Second, by sensitivity analysis, the effect modification of the main effect of exposure $z_{t}$ by x and $r_{t}$ is small. Third, under the assumption of no hidden confounding covariates, we have that

$$\text{causal}\;\text{effect}\;\left(t\right)=E\left(y_{t}\;|\;x,r_{t},\;z_{t}=1\right)-E\left(y_{t}\;|\;x,r_{t\;},\;z_{t}=0\right)=p_{t}\beta_{t},\;t=1,\;2,\;3\text{,}$$

which is a model parameter of the linear model.

Causal effect (4) is an increase in outcome $y_{2}$ during period 2 under the Swedish sequence $\left(z_{1},\;z_{2}\right)=\left(1,\;1\right)$ relative to the common sequence (0,0) during periods 1 and 2. Denote the exposure sequence by $w_{4}$ with $w_{4}=$ 1 for the Swedish sequence $\left(z_{1},\;z_{2}\right)=\left(1,\;1\right)$ and $w_{4}=$ 0 for the common sequence (0,0). The covariates are the population density x and the mortality rate $r_{1}=y_{0}/p_{0}$. With the person weeks $p_{2}$, the regression model for the conditional expectation of $y_{2}$ given ($x,\;r_{1},w_{4})$ is

$$E\left(y_{2}\;|\;x,\;r_{1},w_{4}\right)=p_{2}\left(\alpha_{4}+\gamma_{4}x+\delta_{4}r_{1}+\beta_{4}w_{4}\right).$$

Under the assumption of no hidden confounding covariates, we have that

$$\text{causal}\;\text{effect}\;\left(4\right)=E\left(y_{2}\;|\;x,r_{1},\;w_{4}=1\right)-E\left(y_{2}\;|\;x,r_{1},\;w_{4}=0\right)=p_{2}\beta_{4}\text{.}$$

Causal effect (5) is an increase in outcome $y_{3}$ during period 3 under the Swedish sequence $\left(z_{2},\;z_{3}\right)=\left(1,\;1\right)$ relative to the common sequence (0,0) during periods 2 and 3. Denote the exposure sequence by $w_{5}$ with $w_{5}$ = 1 for the Swedish sequence $\left(z_{2},\;z_{3}\right)=\left(1,\;1\right)$ and $w_{5}$ = 0 for the common sequence (0,0). The covariates are the population density x and the mortality rate $r_{2}=y_{1}/p_{1}$. With the person weeks $p_{3}$, the regression model for the conditional expectation of $y_{3}$ given $\left(x,r_{2},\;w_{5}\right)$ is

$$E\left(y_{3}\;|\;x,r_{2},\;w_{5}\right)=p_{3}\left(\alpha_{5}+\gamma_{5}x+\delta_{5}r_{2}+\beta_{5}w_{5}\right)\text{.}$$

Under the assumption of no hidden confounding covariates, we have that

$$\text{causal}\;\text{effect}\;\left(5\right)=E\left(y_{3}\;|\;x,r_{2},\;w_{5}=1\right)-E\left(y_{3}\;|\;x,r_{2},\;w_{5}=0\right)=p_{3}\beta_{5}$$

Causal effect (6) is an increase in outcome $y_{3}$ during period 3 under the Swedish sequence $\left(z_{1},\;z_{2},\;z_{3}\right)=\left(1,\;1,\;1\right)$ relative to the common sequence (0,0,0) during periods 1, 2 and 3. Denote the exposure sequence by $w_{6}$ with $w_{6}=$1 for the Swedish sequence $\left(z_{1},\;z_{2},\;z_{3}\right)=\left(1,\;1,\;1\right)$ or $w_{6}=0$ for the common sequence (0,0,0). The covariates are the population density x and the mortality rate $r_{1}=y_{0}/p_{0}$. With the person weeks $p_{3}$, the regression model for the conditional expectation of $y_{3}$ given $\left(x,r_{1},\;w_{6}\right)$ is

$$E\left(y_{3}\;|\;x,r_{1},\;w_{6},\right)=p_{3}\left(\alpha_{6}+\gamma_{6}x+\delta_{6}r_{1}+\beta_{6}w_{6}\right)\text{.}$$

Under the assumption of no hidden confounding covariates, we have that

$$\text{causal}\;\text{effect}\;\left(6\right)=E\left(y_{3}\;|\;x,r_{1},\;w_{6}=1\right)-E\left(y_{3}\;|\;x,r_{1},\;w_{6}=0\right)=p_{3}\beta_{6}$$

Based on the probability models and regression models (i.e., the Poisson distribution-based linear regression), we conduct the regressions to yield the estimates, 95% CIs and p-values for causal effects (1)–(6). From the estimated causal effects (1)–(6), we obtain the estimates, 95% CIs and p-values for causal effects (7)–(9) by applying the relationships between causal effects, as described in section 3.4 and Supplementary Materials. For the sake of consistency in evaluating the variability for all causal effects (1)–(9), we use the Monte Carlo simulation to generate 95% CIs and p-values for all these causal effects.

### Estimating causal effects on unemployment

Unemployment refers to the number of unemployed persons aged 15–74, and labor force to the number of both umemployed and employed persons aged 15–74. The unemployment rate is the probability of unemployment among the labor force. Therefore, we assume that conditional on stationary covariates, exposures, and the previous unemployment rate, unemployment follows the binomial distribution with the sample size equal to the labor force and the probability equal to the unemployment rate. The regression models are described below.

Causal effect (1) is an increase in unemployment $y_{2}$ under the Swedish measure $z_{2}=1$ relative to the common measure $z_{2}=0$ during quarter 2. Let $r_{2}=\frac{y_{1}}{p_{1}},$ which is the observed unemployment rate during quarter 1 before exposure $z_{2}$, where $y_{1}$ is the unemployment and $p_{1}$ is the labor force in quarter 1. The covariates are the population density x and the unemployment rate $r_{2}$. With the labor force $p_{2}$ in quarter 2, the regression model for the conditional expectation of $y_{2}$ given $\left(x,r_{2},\;z_{2}\right)$ is

$$E\left(y_{2}\;|\;x,r_{2},\;z_{2}\right)=p_{2}\left(\alpha_{2}+\gamma_{2}x+\delta_{2}r_{2}+\beta_{2}z_{2}\right).$$

Under the assumption of no hidden confounding covariates, we have that

$$\text{causal}\;\text{effect}\;\left(1\right)=E\left(y_{2}\;|\;x,r_{2},\;z_{2}=1\right)-E\left(y_{2}\;|\;x,r_{2},z_{2}=0\right)=p_{2}\beta_{2}\text{.}$$

Causal effect (2) is an increase in unemployment $y_{3}$ under the Swedish measure $z_{3}=1$ relative to the common measure $z_{3}=0$ during quarter 3. Let $r_{3}=y_{2}/p_{2}$, which is the observed unemployment rate during quarter 2 before exposure $z_{3}$. The covariates are the population density x and the unemployment rate $r_{3}$. With the labor force $p_{3}$ in quarter 3, the regression model for the conditional expectation of $y_{3}$ given $\left(x,r_{3},z_{3}\right)$ is

$$E\left(y_{3}\;|\;x,r_{3},z_{3}\right)=p_{3}\left(\alpha_{3}+\beta_{3}z_{3}+\gamma_{3}x+\delta_{3}r_{3}\right)\text{.}$$

Under the assumption of no hidden confounding covariates, we have that

$$\text{causal}\;\text{effect}\;\left(2\right)=E\left(y_{3}\;|\;x,r_{3},z_{3}=1\right)-E\left(y_{3}\;|\;x,r_{3},z_{3}=0\right)=p_{3}\beta_{3}\text{.}$$

Causal effect (3) is an increase in outcome $y_{3}$ during quarter 3 under the Swedish sequence $\left(z_{2},\;z_{3}\right)=\left(1,\;1\right)$ relative to the common sequence (0,0) during quarters 2 and 3. Denote the exposure sequence by $w_{4}$ with $w_{4}=1$ for the Swedish sequence $\left(z_{2},\;z_{3}\right)=\left(1,\;1\right)$ and $w_{4}=0$ for the common sequence (0,0). The covariates are the population density x and the unemployment rate $r_{2}=y_{1}/p_{1}$. With the labor force $p_{3}$ in quarter 3, the regression model for the conditional expectation of $y_{3}$ given $\left(x,r_{2},\;w_{4}\right)$ is

$$E\left(y_{3}\;|\;x,r_{2},\;w_{4}\right)=p_{3}\left(\alpha_{4}+\gamma_{4}x+\delta_{4}r_{2}+\beta_{4}w_{4}\right)$$

Under the assumption of no hidden confounding covariates, we have that

$$\text{causal}\;\text{effect}\;\left(3\right)=E\left(y_{3}\;|\;x,r_{2},\;w_{4}=1\right)-E\left(y_{3}\;|\;x,r_{2},\;w_{4}=0\right)=p_{3}\beta_{4}\text{.}$$

Based on the probability models and regression models (i.e. the binomial distribution-based linear regression), we conduct the regressions to yield the estimates, 95% CIs and p-values for causal effects (1)–(3). From the estimated causal effects (1)–(3), we obtain the estimates, 95% CIs and p-values for causal effect (4) by applying the relationship between causal effects, as described in section 3.5. For the sake of consistency in evaluating the variability for all causal effects (1)–(4), we use the Monte Carlo simulation to generate 95% CIs and p-values for all these causal effects.

## Appendix ASupplementary data

**Supplementary Materials:** It contains (I) the relationships between causal effects, (II) sensitivity analysis for the impact of an alternative follow-up split on the estimation, (III) sensitivity analysis for the impact of population change on the estimation, and (IV) data and code.
