# Supplementary material for: The statistical evidence missing from the Swedish decision-making of COVID-19 strategy during the early period: A longitudinal observational analysis
Source: SSM Popul Health. 2022 Mar 31;18:101083. doi: 10.1016/j.ssmph.2022.101083 (PMC8968210; doi:10.1016/j.ssmph.2022.101083)
Supplement: Multimedia component 1 [file mmc1.docx]

Supplementary Materials for

**The statistical evidence missing from the Swedish decision-making of COVID-19 strategy during the early period: a longitudinal observational analysis**

Xiaoqin Wang, Fan Yang Wallentin and Li Yin

**I. The relationships between causal effects**

For public health outcomes $y_{t}$(general mortality or COVID-19 mortality), exposure $z_{t}$during period $t=1, 2, 3$ takes the Swedish measure ($z_{t}=1)$ or the common measure adopted by the other Nordic countries ($z_{t}=0)$, where $t=1$ for weeks 10-18, $t=$2 for weeks 19-26, $t=$3 for weeks 27-35. The outcome $y_{t}$ is also the covariate for the subsequent exposures $z_{s}$ ($s>t)$. During weeks 1-9, the pandemic had not yet broken out, and no measure was adopted, so there was only the general mortality. Hence, the stationary covariates are population density $x$ and general mortality $y_{0}$.

In causal inference, the sequential causal effect is the causal effect of the exposure sequence relative to the common sequence (adopted by the other Nordic countries) on the outcome during the last period of the sequence ^22,23^. The control sequence is the common sequence. In this study, the length of the exposure sequence is $T=2$or $T=3$. The blip effect of an exposure is the causal effect of the exposure relative to the common measure when setting the subsequent exposures at common measures ^1,2^.

Let $H_{t}=(x,y_{0},z_{1}, y_{1}, \ldots,z_{t-1},y_{t-1})$ be the history of the treatments and outcomes (covariates) up to exposure $z_{t}.$ Given $H_{t}$, let $\boldsymbol{A}_{t}^{T}=(z_{t},\ldots,z_{T})$ be the exposure sequence and $\boldsymbol{B}_{t}^{T}=\boldsymbol{0}_{t}^{T}=(0,\ldots, 0)$ the common sequence(the control sequence). Let $y_{T}\left( \boldsymbol{A}_{t}^{T} \right)$ be the potential outcome during period $T$ under exposure sequence $\boldsymbol{A}_{t}^{T}$ and $y_{T}\left( \boldsymbol{0}_{t}^{T} \right)$ under $\boldsymbol{0}_{t}^{T}$. Then the sequential causal effect of $\boldsymbol{A}_{t}^{T}$ relative to $\boldsymbol{0}_{t}^{T}$ given history $H_{t}$ is equal to

$$\text{SCE(}\boldsymbol{A}_{t}^{T} \left| H_{t} \right)=E\left\{ y_{T}\left( \boldsymbol{A}_{t}^{T} \right) | H_{t} \right\}-E\left\{ y_{T}\left( \boldsymbol{0}_{t}^{T} \right) | H_{t} \right\}.$$

Clearly, $\text{SCE(}\boldsymbol{B}_{t}^{T}|H_{t})=0$. The blip effect of the exposure $z_{s}$ given history $H_{s}$is equal to

$$\phi\left( z_{s} | H_{s} \right)=E\left\{ y_{T}\left( {z_{s}\boldsymbol{,0}}_{s+1}^{T} \right) | H_{s} \right\}-E\left\{ y_{T}\left( \boldsymbol{0}_{s}^{T} \right) | H_{s} \right\}.$$

Clearly, $\phi\left( z_{s}=0 | H_{s} \right)=0$.

Please notice that in Wang and Yin^2^, the sequential causal effect of $\boldsymbol{A}_{1}^{T}$ relative to $\boldsymbol{B}_{1}^{T}$ in the population is denoted by $\text{SCE(}\boldsymbol{A}_{1}^{T}; \boldsymbol{B}_{1}^{T})$. Following their logic, the sequential causal effect of $\boldsymbol{A}_{t}^{T}$ relative to $\boldsymbol{B}_{t}^{T}$ given history $H_{t}$ should be denoted by $\text{SCE(}\boldsymbol{A}_{1}^{T}; \boldsymbol{B}_{1}^{T} | H_{t})$. But here, it is denoted by $\text{SCE(}\boldsymbol{A}_{t}^{T} | H_{t})$. Furthermore, the blip effect is denoted by $\phi\left( H_{t}; z_{t} \right)$. But here it is denoted by $\phi\left( z_{t} |H_{t} \right).$Therefore, by applying formula (18) in Theorem 2 of Wang and Yin^2^ and $\phi\left( z_{s}=0 |H_{s} \right)=0$, we obtain

$$\text{SCE(}\boldsymbol{A}_{t}^{T} |H_{t})=\phi\left( z_{t} | H_{t} \right)+\sum_{s=t+1}^{T} E\{\phi\left( z_{s} | H_{s} \right)\}$$

where the expectation is with respect to the $\prod_{t}^{s-1} \text{pr}\left( y_{k} | H_{k}{,z}_{k} \right).$

As described in Appendix, we only consider the main effect of the exposure. Thus, we assume that $\text{SCE(}\boldsymbol{A}_{t}^{T} \left| H_{t} \right)$ and $\phi\left( z_{s} | H_{s} \right)$ do not vary with the history, i.e.,

$\text{SCE(}\boldsymbol{A}_{t}^{T} | H_{t})=\text{SCE(}\boldsymbol{A}_{t}^{T}), \phi\left( z_{s} |H_{s} \right)=\phi\left( z_{s} \right)$.

Then we have $E\{\phi\left( z_{s} | H_{s} \right)\}=\phi\left( z_{s} \right)$ and the above formula is simplified into

$$\text{SCE(}\boldsymbol{A}_{t}^{T})=\phi\left( z_{t} \right)+\ldots+\phi\left( z_{T} \right), t=1, 2, \ldots T. (I)$$

Noticeably, we still need to adjust for the history when estimating the main effect. By applying the assumption of no hidden confounding covariate described in Section 2.3, we only need to adjust for the shortened history $H_{t}=(x,y_{t-1})$ in the regression model as described in Appendix.

Now we consider the case of $T=2$ with $\boldsymbol{A}_{1}^{2}=$ ${(z}_{1}, z_{2})=(1, 1)$, which is the Swedish sequence during periods 1 and 2. Then the outcome is $y_{2}$ under period $T=2$ and we have sequential causal effect $\text{SCE(}\boldsymbol{A}_{1}^{2})$ as well as the blip effects $\phi\left( z_{1}=1 \right)$and $\phi\left( z_{2}=1 \right)$ on $y_{2}$. The above formula becomes

$$\text{SCE(}\boldsymbol{A}_{1}^{2})=\phi\left( z_{1}=1 \right)+\phi\left( z_{2}=1 \right). (\mathrm{II})$$

On the other hand, the sequential causal effect is

$\text{SCE(}\boldsymbol{A}_{1}^{2})=E\left\{ y_{2}\left( \boldsymbol{A}_{1}^{2} \right) | H_{1} \right\}-E\left\{ y_{2}\left( \boldsymbol{0}_{1}^{2} \right) | H_{1} \right\},$

Here the expectations $E\left\{ y_{2}\left( \boldsymbol{A}_{1}^{2} \right) | H_{1} \right\}$ and $E\left\{ y_{2}\left( \boldsymbol{0}_{1}^{2} \right) | H_{1} \right\}$ may depend on the history $H_{1}$, though $\text{SCE(}\boldsymbol{A}_{1}^{2})$ does not. In statistical practices, it is a commonplace that the means depend on the history, whereas the difference between the means (i.e., the effect) does not. Here we see that$\text{ SCE(}\boldsymbol{A}_{1}^{2})$ is an increase in outcome $y_{2}$ during period 2 under the Swedish sequence relative to the common sequence during periods 1 and 2, i.e., causal effect (4). Therefore we have

$$\text{SCE(}\boldsymbol{A}_{1}^{2})=\text{causal effect (4)}$$

The blip effect of $z_{1}$ is

$$\phi\left( z_{1}=1 \right)=E\left\{ y_{2}\left( 1, 0 \right) | H_{1} \right\}-E\left\{ y_{2}\left( \boldsymbol{0}_{1}^{2} \right) | H_{1} \right\},$$

where $E\left\{ y_{2}\left( 1, 0 \right) | H_{1} \right\}$ and $E\left\{ y_{2}\left( \boldsymbol{0}_{1}^{2} \right) | H_{1} \right\}$ may depend on the history $H_{1}$ but $\phi\left( z_{1}=1 \right)$ does not. Here we see that $\phi\left( z_{1}=1 \right)$ is an increase in outcome $y_{2}$ during period 2 under the mixed sequence $(1, 0)$ relative to the common sequence $(0, 0)$ during periods 1 and 2, i.e., causal effect (7). Therefore we have

$$\phi\left( z_{1}=1 \right)=causal effect (7).$$

The blip effect of $z_{2}$ is

$$\phi\left( z_{2}=1 \right)=E\left\{ y_{2}\left( 1 \right) | H_{2} \right\}-E\left\{ y_{2}\left( 0 \right) | H_{2} \right\},$$

which is an increase in outcome $y_{2}$ under the Swedish measure relative to the common measure during period 2, i.e., causal effect (2). Therefore we have

$$\phi\left( z_{2}=1 \right)=causal effect (2).$$

Now by applying formula (II) above, we obtain the following relationship between causal effects

$$\text{causal effect (4) }=\text{causal effect (7) }+\text{causal effect (2)}\text{,}$$

which is the equality used to obtain the estimate of $\text{causal effect (7)}$ in section 3.4 of the main text.

Now we consider the case of $T=3$. Then we have two Swedish sequences: $\boldsymbol{A}_{2}^{3}=$ $(z_{2}, z_{3})=(1, 1)$ during periods 2, and 3 and $\boldsymbol{A}_{1}^{3}=$ ${(z}_{1}, z_{2}, z_{3})=(1, 1, 1)$ during periods 1, 2, and 3. The outcome is $y_{3}$ during period $T=3$. We have two sequential causal effects$\text{ }\text{SCE(}\boldsymbol{A}_{2}^{3})$ and $\text{SCE(}\boldsymbol{A}_{1}^{3})$ as well as three blip effects $\phi\left( z_{1}=1 \right),$ $\phi\left( z_{2}=1 \right)$ and $\phi\left( z_{3}=1 \right)$ on $y_{3}$. Please notice that the blip effects here are on the outcome $y_{3}$and not the same as the blip effects in the case of $T=2,$ which are on the outcome $y_{2}$. We use the same notation for notational simplicity. In this case, the formula (I) at $t=2$ becomes

$$\text{SCE(}\boldsymbol{A}_{2}^{3})=\phi\left( z_{2}=1 \right)+\phi\left( z_{3}=1 \right) (\mathrm{III})$$

and at $t=1$ becomes

$$\text{SCE(}\boldsymbol{A}_{1}^{3})=\phi\left( z_{1}=1 \right)+\phi\left( z_{2}=1 \right)+\phi\left( z_{3}=1 \right). (\mathrm{IV})$$

On the other hand, the sequential causal effect

$$\text{SCE(}\boldsymbol{A}_{2}^{3})=E\left\{ y_{3}\left( \boldsymbol{A}_{2}^{3} \right) | H_{2} \right\}-E\left\{ y_{3}\left( \boldsymbol{0}_{2}^{3} \right) | H_{2} \right\}$$

is an increase in outcome $y_{3}$ during period 3 under the Swedish sequence relative to the common sequence during periods 2 and 3, i.e., causal effect (5). Therefore we have that

$$\text{SCE(}\boldsymbol{A}_{2}^{3})=causal effect (5).$$

The blip effect of $z_{2}$

$\phi\left( z_{2}=1 \right)=E\left\{ y_{3}\left( 1, 0 \right) | H_{2} \right\}-E\left\{ y_{3}\left( \boldsymbol{0}_{2}^{3} \right) | H_{2} \right\}$

is an increase in outcome $y_{3}$ during period 3 under the mixed sequence $(1, 0)$ relative to the common sequence $(0, 0)$ during periods 2 and 3, i.e., causal effect (8). Therefore we have that

$$\phi\left( z_{2}=1 \right)=causal effect \left( 8 \right).$$

The blip effect of $z_{3}$

$$\phi\left( z_{3}=1 \right)=E\left\{ y_{3}\left( 1 \right) | H_{3} \right\}-E\left\{ y_{3}\left( 0 \right) | H_{3} \right\}$$

is an increase in outcome $y_{3}$ under the Swedish measure relative to the common measure during period 3, i.e., the causal effect (3). Therefore we have

$$\phi\left( z_{3}=1 \right)= causal effect (3).$$

Now by applying formula (III) above, we obtain the following relationship between causal effects

$$\text{causal effect (5) }=\text{causal effect (8) }+\text{causal effect (3),}$$

which is the equality used to estimate $\text{causal effect (8)}$ in section 3.4 of the main text.

Furthermore, the sequential causal effect

$$\text{SCE(}\boldsymbol{A}_{1}^{3})=E\left\{ y_{3}\left( \boldsymbol{A}_{1}^{3} \right) | H_{1} \right\}-E\left\{ y_{3}\left( \boldsymbol{0}_{1}^{3} \right) | H_{1} \right\}$$

is an increase in $y_{3}$ during period 3 under the Swedish sequencerelative to the common sequence during periods 1, 2, and 3, i.e., causal effect (6). Therefore we have that

$$\text{SCE(}\boldsymbol{A}_{1}^{3})=causal effect (6).$$

The blip effect of $z_{1}$

$$\phi\left( z_{1}=1 \right)=E\left\{ y_{3}\left( 1, 0, 0 \right) |H_{1} \right\}-E\left\{ y_{3}\left( \boldsymbol{0}_{1}^{3} \right) | H_{1} \right\}$$

is an increase in outcome $y_{3}$ during period 3 under the mixed sequence $(1, 0, 0)$ relative to the common sequence $(0, 0, 0)$ during periods 1, 2, and 3, i.e., causal effect (9). Therefore we have

$$\phi\left( z_{1}=1 \right)=\text{causal effect (9).}$$

Now by applying formula (IV) above, we obtain the following relationship between causal effects

$$\text{causal effect (6) }=\text{causal effect (9) }+\text{causal effect (8) }+\text{causal effect (3).}$$

which is the equality used to obtain the estimate of $\text{causal effect (9)}$ in section 3.4 of the main text.

**II. Sensitivity analysis for the impact of an alternative follow-up split on the estimation**

A summary of population density, exposures, outcomes, and the follow-up is given in Table 2. As described in Section 2.3, the assumption of no hidden confounding covariates is: given the population density and outcome in a period, no other covariates exist that confound the causal effects of exposures in the subsequent periods. As described in Appendix, we estimate causal effects (1)-(6) with the assumption and the data by the regression. From the estimated causal effects, we obtain the estimates for causal effects (7)-(9) by applying the relationships between causal effects described above. In Tables 3, 4, and 5, we present the estimates of these causal effects.

In the analysis, we split the entire follow-up into weeks 1-9, 10-18, 19-26, and 27-35. Now, we make small changes to the splitting method and split the entire follow-up into weeks 1-9, 10-17, 18-26, and 27-35. Then we conducted the same analysis for COVID-19 mortality and general mortality.

The results are presented in Table S1 for the two splitting methods. This table shows that the estimated causal effects are slightly different between the two splitting methods. Therefore, the conclusions from the two splitting methods are the same.

**III. Sensitivity analysis for the impact of population change on the estimation**

When estimating the causal effects on COVID-19 mortality and general mortality, we need to calculate the person weeks $p_{0}$, $p_{1}$,$p_{2}$, and $p_{3},$ where weeks are the numbers of weeks during a period, and persons are the population size during the same period. We use the population size of December 2019 for all the estimations. This approximation is usually reasonable because general mortality is far smaller than the population size. On the other hand, there is a considerable increase in general mortality during the pandemic and thus a reduction in the population size. To examine the impact of population change, we conduct a sensitivity analysis, in which we subtract the population size by general mortality during earlier periods and then use the obtained population size to estimate the causal effect on the general mortality during the current period.

The results are presented in Table S2 together with those obtained with the population size of December 2019. As shown in this table, the causal effects estimated with the two population sizes are nearly identical. Therefore, we conclude that the change of population size by COVID-19 mortality has little impact on the estimation of the causal effect.

**IV. Data and code**

Data and code for the analysis are included as a separate file (data and code.zip). The software used is SAS 9.4 (SAS Institute Inc., Cary, NC, USA).

**References:**

1. M. A. Hernan and J. M. Robins, Causal Inference: What If (Chapman & Hall/CRC, Boca Raton, 2020).
2. X. Wang and L. Yin, New G-Formula for the Sequential Causal Effect and Blip Effect of Treatment in Sequential Causal Inference. *Annals of Statistics* **48**, 138-160 (2020)

Table S1. Sensitivity analysis for the impact of alternative follow-up split.

Causal effects (1)-(9): defined in Tables 3, 4, and 5 and measured per 100,000 individuals.

Outcome: general mortality or COVID-19 mortality.

Two follow-up splits: (a) weeks 1-9, 10-18, 19-26, 27-35 (used in the article); (b) weeks 1-9, 10-17, 18-26, 27-35.

| $\left( \begin{matrix} \mathrm{Estimate} \\ 95\% CI \\ \text{p-}\mathrm{value} \end{matrix} \right)$ for the causal effect estimated with two splitting methods | | | | |
| --- | --- | --- | --- | --- |
| Causal effect | Split (a) | | Split (b) | |
|  | General mortality | COVID-19 mortality | General mortality | COVID-19 mortality |
| (1) | 20.2  (16.6, 23.7)  < 0.001 | 18.7  (17.6, 19.8)  < 0.001 | 16.4  (13.1, 19.8)  < 0.001 | 13.5  (12.5, 14.5),  < 0.001 |
| $\left( 2 \right)$ | $-$2.2  ($-$7.4, 3.1)  0.419 | 14.4  (12.8, 16.0)  < 0.001 | 0.0  ($-$6.0, 6.1)  0.987 | 18.1  (16.3, 19.9)  < 0.001 |
| (3) | $-$17.6  ($-$22.5, $-$12.6)  < 0.001 | 1.9  (0.5, 3.3)  0.007 | $-$18.8  ($-$23.7, $-$13.9)  < 0.001 | 2.0  (0.6, 3.4)  0.006 |
| (4) | 11.9  (8.6, 15.2)  < 0.001 | 20.3  (19.3, 21.2)  < 0.001 | 15.6  (12.1, 19.1)  < 0.001 | 25.2  (24.1, 26.4)  < 0.001 |
| (5) | $-$17.4  ($-$22.1, $-$12.6)  < 0.001 | 3.3  (2.7, 4.0)  < 0.001 | $-$16.6  ($-$21.5, $-$11.9)  < 0.001 | 3.4  (2.7, 4.0)  < 0.001 |
| (6) | $-$7.3  ($-$10.6, $-$4.0)  < 0.001 | 3.15  (2.8, 3.5)  < 0.001 | $-$7.3  ($-$10.6, $-$4.0)  < 0.001 | 3.2  (2.8, 3.5)  < 0.001 |
| (7) | 14.0  (10.2, 17.9)  < 0.001 | 5.9  (4.4, 7.3)  < 0.001 | 15.5  (10.9, 20.2)  < 0.001 | 7.1  (5.5, 8.7)  < 0.001 |
| (8) | 0.2  ($-$4.9, 5.2)  0.947 | 1.4  (0.2, 2.7)  0.024 | 2.1  ($-$2.9, 7.2)  0.406 | 1.4  (0.1, 2.6)  0.034, |
| (9) | 10.1  (6.6, 13.6)  < 0.001 | $-$0.2  ($-$0.8, 0.4)  0.505 | 9.4  (5.7, 13.1)  < 0.001 | $-$0.2  ($-$0.7, 0.3)  0.383 |

Table S2. Sensitivity analysis for the impact of population change caused by general mortality.

Causal effects (1)-(9): defined in Tables 3, 4, and 5 and measured per 100,000 individuals.

Outcome: general mortality.

Two population sizes: (a) population size of December 2019 (used in the article); (b) population size of December 2019 subtracted by general mortality in earlier periods

| $\left( \begin{matrix} \mathrm{Estimate} \\ 95\% CI \\ \text{p-}\mathrm{value} \end{matrix} \right)$ for the causal effect estimated with two population sizes | | |
| --- | --- | --- |
| Causal effect | Population (a) | Population (b) |
| (1) | 20.2  (16.6, 23.7)  < 0.001 | 20.2  (16.6, 23.8)  < 0.001 |
| (2) | $-$2.2  ($-$7.4, 3.1)  0.419 | $-$2.2  ($-$7.5, 3.0)  0.418 |
| (3) | $-$17.6  ($-$22.5, $-$12.6)  < 0.001 | $-$17.6  ($-$22.6, $-$12.6)  < 0.001 |
| (4) | 11.9  (8.6, 15.2)  < 0.001 | 11.9  (8.6, 15.2)  < 0.001 |
| (5) | $-$17.4  ($-$22.1, $-$12.6)  < 0.001 | $-$17.5  ($-$22.2, $-$12.7)  < 0.001 |
| (6) | $-$7.3  ($-$10.6, $-$4.0)  < 0.001 | $-$7.3  ($-$10.6, $-$4.0)  < 0.001 |
| (7) | 14.0  (10.2, 17.9)  < 0.001 | 14.1  (10.2, 17.9)  < 0.001 |
| (8) | 0.2  ($-$4.9, 5.2)  0.947 | 0.2  ($-$4.9, 5.3)  0.942 |
| (9) | 10.1  (6.6, 13.6)  < 0.001 | 10.2  (6.6, 13.7)  < 0.001 |
